# H_2_ Uptake and Diffusion Characteristics in Sulfur-Crosslinked Ethylene Propylene Diene Monomer Polymer Composites with Carbon Black and Silica Fillers after High-Pressure Hydrogen Exposure Reaching 90 MPa

**DOI:** 10.3390/polym15010162

**Published:** 2022-12-29

**Authors:** Jae Kap Jung, Ji Hun Lee, Sang Koo Jeon, Un Bong Baek, Si Hyeon Lee, Chang Hoon Lee, Won Jin Moon

**Affiliations:** 1 Hydrogen Energy Materials Research Center, Korea Research Institute of Standards and Science, Daejeon 34113, Republic of Korea; 2 Department of Biochemical and Polymer Engineering, Chosun University, Gwangju 61452, Republic of Korea; 3 Gwangju Center, Korea Basic Science Institute, Gwangju 61186, Republic of Korea

**Keywords:** carbon black, silica, H_2_ uptake, diffusion, permeation, density, dispersion

## Abstract

We investigated the influence of two fillers—CB (carbon black) and silica—on the H_2_ permeation of EPDM polymers crosslinked with sulfur in the pressure ranges 1.2–90 MPa. H_2_ uptake in the CB-blended EPDM revealed dual sorption (Henry’s law and Langmuir model) when exposed to pressure. This phenomenon indicates that H_2_ uptake is determined by the polymer chain and filler-surface absorption characteristics. Moreover, single sorption characteristics for neat and silica-blended EPDM specimens obey Henry’s law, indicating that H_2_ uptake is dominated by polymer chain absorption. The pressure-dependent diffusivity for the CB-filled EPDM is explained by Knudsen and bulk diffusion, divided at the critical pressure region. The neat and silica-blended EPDM specimens revealed that bulk diffusion behaviors decrease with decreasing pressure. The H_2_ diffusivities in CB-filled EPDM composites decrease because the impermeable filler increases the tortuosity in the polymer and causes filler–polymer interactions; the linear decrease in diffusivity in silica-blended EPDM was attributed to an increase in the tortuosity. Good correlations of permeability with density and tensile strength were observed. From the investigated relationships, it is possible to select EPDM candidates with the lowest H_2_-permeation properties as seal materials to prevent gas leakage under high pressure in H_2_-refueling stations.

## 1. Introduction

The fundamental properties of rubbery polymers can be improved by blending fillers. Fillers in polymer composites achieve multiple purposes, the most important including reinforcement, improvement in processing, diffusing molecule impermeability, an increase in oil resistance, and a reduction in material cost [1,2,3,4,5,6,7]. The rubber industry uses a wide range of fillers because of their merits in rubber compounding. In particular, carbon black (CB) filler compounding with rubber enhances the mechanical properties of rubber composites, such as hardness, tear strength, tensile strength, modulus, and abrasive strength [8,9,10]. The size and surface area of CB filler particles are important factors affecting reinforcements in rubbers [11]. Furthermore, the reinforcement originates from filler–filler and rubber–filler interactions.

Among nonblack fillers, silica filler provides unique strength characteristics that enhance the abrasion resistance, tear strength, aging resistance, and adhesion properties of rubber [12,13]. Precipitated silica provides the highest degree of reinforcement. Silane coupling agents enhance the chemical compatibilities of silica fillers with the rubber matrix for more efficient reinforcement [14,15,16].

Several studies have reported the influence of filler loading on gas transport properties in polymers, which is associated with the consecutive processes of sorption, diffusion, and desorption in the polymer membrane; these processes determine the solubility, diffusion coefficient, and permeation coefficient. The diffusion characteristics of ethyl *p*-aminobenzoate for silicone rubber membranes containing various amounts of fumed silica filler with high surface areas were described [17]. Increased filler loading results in a decrease in the transmission rate but this apparent diffusivity decreases drastically due to the adsorption of the permeant at the filler. This result is attributed to the adsorption of permeant at the filler surface.

Ordobina et al. [18] reported that crosslinked filler particles in poly(butyl methacrylate) latex films can either enhance or delay the diffusion rate. The effect of soft filler particles on polymer diffusion is a combination of an obstacle effect, delaying polymer diffusion, and a second effect, probably a free-volume effect that promotes polymer diffusion. The change in the diffusion properties of the polymer membrane by the presence of fillers is associated with the fact that polymer chains near a filler surface have different properties than those in the bulk state. This phenomenon occurs due to the chain conformational changes attributed to the presence of a boundary and interaction between the polymer and the filler.

Moreover, the filler effect on the mechanical properties of natural rubber blended with different CB contents have been investigated. Tensile strength and hardness increased with the addition of CB. When the size of the CB particles was small, it formed a significant interaction with the natural rubber matrix [19]. The decrease in the molecular size of the CB filler generally improved the mechanical properties [20]. When the CB filler entered the rubber, the flexibility of the rubber chain decreased, resulting in a more rigid vulcanizate. This rigidity originates from filler–filler and rubber–filler interactions, occurring at various length scales due to the CB structure [21,22,23].

According to a previous investigation into filler influence [24], the N_2_ surface area of CB slightly affected H_2_ permeation in filled EPDM and obviously influenced the H_2_ diffusion coefficient and solubility. The CB-filled EPDM showed a decreased diffusivity, and its solubility increased with increasing N_2_ surface area because the H_2_ molecules were adsorbed by the CB participate in diffusion. H_2_ solubility in the filled EPDM is further influenced by the filler surface area and the interfacial structure between the filler and the polymer network. Similar studies of the filler effects on H_2_ permeability were systematically conducted for nitrile butadiene rubber (NBR) [25] and EPDM [26] composites. In these studies, the contents of different fillers varied up to 60 phr. The CB and silica fillers were found to decrease H_2_ diffusivity and permeation and H_2_ solubility and diffusivity depended on the filler type.

Based on previous investigations, the present study contains an EPDM composite commonly used as an O-ring in gas seals under high pressure up to 90 MPa at H_2_-refueling stations [27]. The study is associated with the H_2_ transport properties of sulfur-crosslinked EPDM blended with CB and silica fillers. By investigating the influence of filler loadings on H_2_ gas permeability in these composites, we reveal the related sorption, diffusion phenomena, and the filler-induced permeation properties of the composites. The H_2_ permeation characteristics of the polymer compounds were measured precisely using a modified volumetric analysis technique (VAT) and a diffusion analysis algorithm [28,29]. The H_2_ uptakes, solubilities, diffusion coefficients, and permeabilities of the EPDM composites blended with three filler types were investigated regarding exposure pressure, filler contents, and filler types. The aims of this study were to determine the pressure-dependent H_2_ sorption and diffusion mechanisms, to find possible correlations between the compositions and bulk properties of the materials, and to identify the dominant effects of permeation in the filled EPDM composites.

## 2. Materials and Methods

### 2.1. Sample Composition

Table 1 shows the formulations of the EPDM compounds: one neat EPDM without filler, six EPDM specimens with CB filler, and three EPDM specimens with silica filler. Two types of CB filler were used—a high abrasion furnace (HAF) CB and a semi-reinforcing furnace (SRF) CB—with particle sizes of 32 and 65 nm, respectively, and specific surface areas of 76 and 30 m^2^/g, respectively. The specific surface area of silica was 175 m^2^/g. The EPDM composites blended with filler are designated EPDM HAFa, EPDM SRFb, and EPDM Sc; a, b, and c denote the parts per hundred rubber components (phr) of HAF, SRF, and silica, respectively. For instance, EPDM HAF20 represents EPDM blended with HAF CB with 20 phr. Sulfur with 1.5 phr is used as a crosslinking agent. The compounding method for EPDM rubber is detailed in the literature [26].

### 2.2. Exposure to H_2_ Gas

The high-pressure chamber and purge conditions used in this work are described elsewhere [29]. Cylindrical rubber samples with diameters of 13 mm and thicknesses of 3 mm were exposed to hydrogen gas for more than 20 h at pressures ranging from 1.2 to 90 MPa. After exposure to high-pressure H_2_, the chamber valve was opened to emit the H_2_ gas. After decompression, a specimen was loaded into a graduated cylinder. The amount of H_2_ gas released during the lag time was determined by measuring the offset value using a diffusion analysis program [29].

### 2.3. Transmission Electron Microscopy

The microstructures of the EPDM specimens were investigated with a combination of focused ion beam (FIB) and transmission electron microscopy (TEM). Thin foil samples for TEM image observation were prepared with an FIB. The morphology, distribution, and size characteristics of the CB filler particles in EPDM were observed with a transmission electron microscope (TECNAI F20, FEI company, Hillsboro, OR, USA) operating at an accelerating voltage of 200 kV.

## 3. Measurement Method and Diffusion Analysis Program

### 3.1. Volumetric Measurement for Emitting H_2_

The volumetric measurement utilized the graduated cylinders in which the emitted H_2_ from the specimen was collected and measured. After exposure in the high-pressure chamber and subsequent decompression, the samples were obtained. The samples were loaded into their corresponding gas cell spaces at the top of the graduated cylinder. The details for the method are comprehensively described elsewhere [28,29].

To obtain the increased number of moles (Δ*n*) due to the hydrogen gas released in the graduated cylinder, we measured the volume increase (Δ*V*); that is, we measured the reduction in the water level in the cylinder as follows [29]:

(1) $$\Delta n=\frac{(P_{o}-\rho gh)\Delta V}{RT}$$

where *ρ* is the distilled water density, *g* is the gravitational acceleration, *h* is the height (vertical position) of the water level in the cylinder measured from the water level in the corresponding water container, and *P_0_* is the atmospheric pressure outside the cylinder.

The Δ*n* in the cylinder is converted to the mass concentration [*C*(*t*)] of H_2_ released from the rubber sample:

(2) $$C\left(t\right)\left[\mathrm{wtppm}\right]=\Delta n\left[\mathrm{mol}\right]\times \frac{m_{H2}\;\left[\frac{g}{\mathrm{mol}}\right]}{m_{sample}\left[g\right]}\times 10^{6}$$

where $m_{H2}$ [*g*/mol] is the H_2_ molar mass, which is equal to 2.016 g/mol and $m_{sample}$ is the sample mass.

### 3.2. Diffusion Analysis Program

Assuming that H_2_ desorption is a Fickian diffusion process, the concentration $C_{E}\left(t\right)$ of the emitted H_2_ is computed as follows [30,31]:

(3) $$\begin{array}{l} \frac{C_{E}\left(t\right)}{C_{\infty }}=1-\frac{32}{\pi^{2}}\times \left[\overset{\infty }{\underset{n=0}{\sum }}\frac{exp\left\{\frac{-{\left(2n+1\right)}^{2}\pi^{2}Dt}{l^{2}}\right\}}{{\left(2n+1\right)}^{2}}\right]\times \left[\overset{\infty }{\underset{n=1}{\sum }}\frac{exp\left\{-\frac{D\beta_{n}^{2}t}{\rho^{2}}\right\}}{\beta_{n}^{2}}\right]\\ \;=1\\ \;-\frac{32}{\pi^{2}}\times \left[\frac{\exp\left(-\frac{\pi^{2}Dt}{l^{2}}\right)}{1^{2}}+\frac{\exp\left(-\frac{3^{2}\pi^{2}Dt}{l^{2}}\right)}{3^{2}}+\ldots ,+\frac{\exp\left(-\frac{{\left(2n+1\right)}^{2}\pi^{2}Dt}{l^{2}}\right)}{{\left(2n+1\right)}^{2}}+\ldots ,\right]\\ \;\times \left[\frac{\exp\left(-\frac{D\beta_{1}^{2}t}{\rho^{2}}\right)}{\beta_{1}^{2}}+\frac{\exp\left(-\frac{D\beta_{2}^{2}t}{\rho^{2}}\right)}{\beta_{2}^{2}}+\ldots ,+\frac{\exp\left(-\frac{D\beta_{n}^{2}t}{\rho^{2}}\right)}{\beta_{n}^{2}}+\ldots ,\right] \end{array}$$

where *β_n_* is the root of the zeroth order Bessel function *J_0_(β*_n_*)* with *β*_1_ = 2.40483, *β*_2_ = 5.52008, *β*_3_ = 8.65373, …, *β*_50_ = 156.295. Equation (3) is an infinite series expansion with two summations. This equation is a solution for Fick’s second diffusion equation for a cylindrical-shaped sample. In this study, *C_E_* = 0 at *t* = 0 and *C_E_* =$\;C_{\infty }$ at *t* = ∞. $C_{\infty }$ is the saturated H_2_ concentration at infinity, i.e., the H_2_ uptake. *D* is the diffusion coefficient, and l and ρ are the thickness and radius of the cylindrical specimen, respectively.

The derivative at t=0, ($\frac{dC_{E}\left(t=0\right)}{dt}$), of two summations in Equation (3) is −∞. This finding implies that the initial H_2_ emission rate is extremely fast; it originates from the distribution difference of H_2_ caused by the discontinuous pressure difference between the high pressure inside the specimen and the atmosphere outside the specimen after decompression. This result means that, according to Equation (3), there is a possibility of showing different evolution characteristics with time just after decompression.

**Figure 1:**
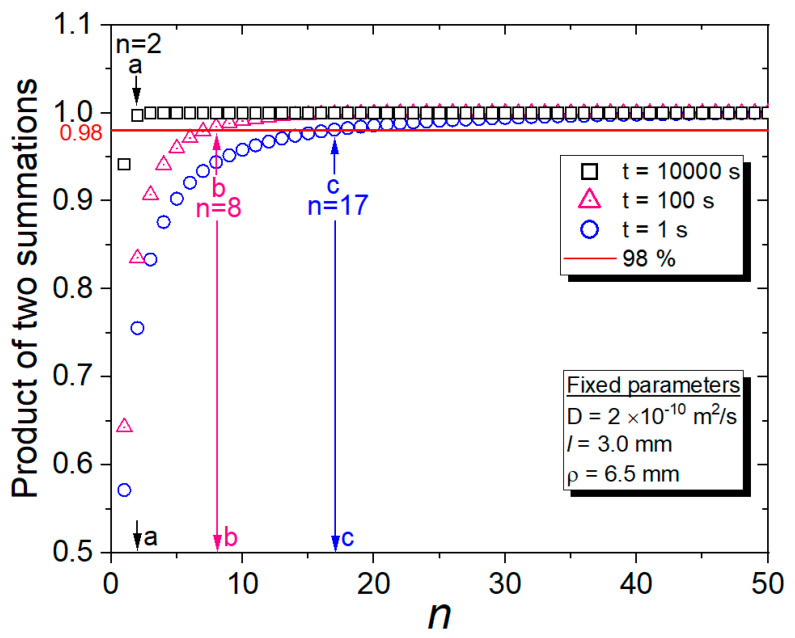
Product of two summations vs. *n* at different times.

Because Equation (3) has two infinite terms, we estimate that the finite number of terms (*n*) in the actual calculation of the two summations should be contained to obtain *D* and $C_{\infty }$. Thus, we calculate the contributions in two summations of Equation (3), reaching *n* = 50 terms with three different times *t*. Figure 1 shows the normalized and calculated product of two summations versus *n* at three different times (*t* = 1 s, 100 s, and 10,000 s) at fixed parameters of *l* = 3.0 mm, *ρ* = 6.5 mm, and *D* = 2 × 10^−10^ m^2^/s. With increasing *n* up to 50, the products of the two summations for all *t* = 1 s, 100 s, and 10,000 s converge to 1 (the value when *n* is infinite). The horizontal red line in Figure 1 corresponds to a 0.98 value of converged value 1. The corresponding *n*-term value on the *x*-axis exceeds 0.98 on the *y*-axis, for which *n* should be contained in two summations as nearly equal to the converged value of 1. Thus, a, b, and c, which are obtained from the intersection between the 98% line and the product of two summations (data on *y*-axis), indicate the minimum number of terms, *n*, to be included in the summations; these values correspond to 2 at *t* = 10,000 s, 8 at *t* = 100 s, and 17 at *t* = 1 s, respectively, in *n*. When *t* is sufficiently greater than 10,000 s, the two terms of the two summations in Equation (3) mainly contribute to the $C_{E}\left(t\right)$ value. However, if t is less than t=100 s, Equation (3) cannot easily converge, and eight more *n* terms are needed, as shown by arrow b in Figure 1. For the calculation with an uncertainty less than 2% using Equation (3), we should include many terms that are greater than the *n* = 64 terms (8 × 8) in the product of the two summations at *t* = 100 s. Thus, a dedicated calculation program is needed. We developed a diffusion analysis program to calculate *D* and $C_{\infty }$, including up to *n* = 100 terms of the first summation and *n* = 50 terms of the second summation, reaching $\beta_{50}$ in Equation (3) for covering small time values, *t* = 1 s.

**Figure 2:**
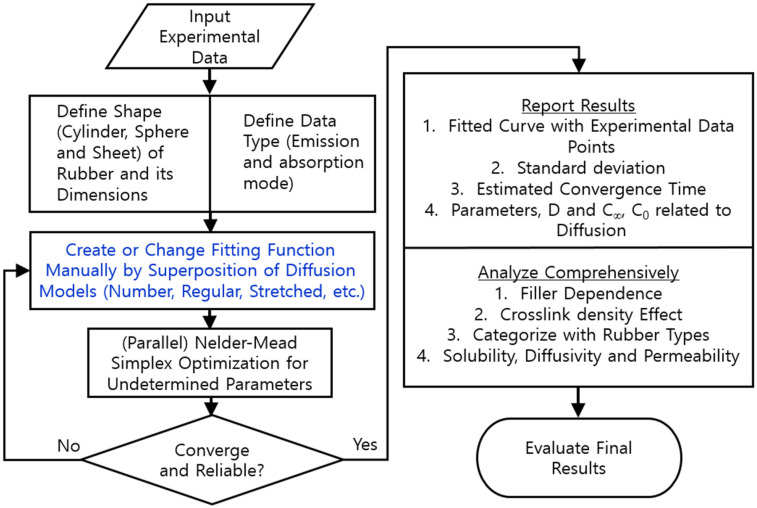
Flowchart for analyzing the diffusion data set for various types of rubbers via the Nelder–Mead simplex nonlinear optimization method.

Figure 2 shows the overall flowchart of the diffusion analysis program developed to analyze the $C_{E}\left(t\right)$ data using Equation (3) by a Nelder–Mead simplex nonlinear optimization algorithm [32]. As a result of the application of the developed program, the solubility, diffusivity, and permeability were finally obtained in a rubber composite system. The contents indicated by the blue color in Figure 2 are related to the selection of the solution to the diffusion equation of Equation (3); for this equation, we chose an appropriate diffusion model corresponding to the rubber shape and the number of superposition models.

The *D* and $C_{\infty }$ for EPDM composites were determined by utilizing a diffusion analysis program based on Figure 1 and Figure 2. An example application for the analysis program and the detailed procedure were already described in the previous literature [29]. The method of restoring H_2_ content using the diffusion analysis program is regarded as a novel aspect of our research. The precise measurement of H_2_ content, in particular, is possible as a result of including up to *n* = 100 terms in the summations with the help of the diffusion analysis program.

## 4. Results and Discussion

### 4.1. TEM

**Figure 3:**
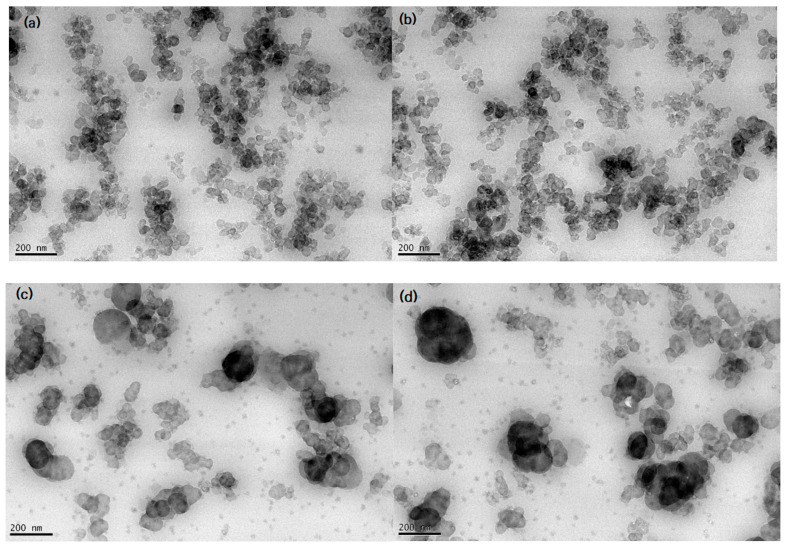
TEM image showing CB particles and aggregates in the CB-filled EPDM composite with (**a**,**b**) HAF20, and (**c**,**d**) SRF20. (**a**,**b**) were taken at different parts of the EPDM HAF20 specimen. (**c**,**d**) were taken at different parts of the EPDM SRF20 specimen.

Figure 3a–d shows the TEM micrographs of EPDM HAF20 and SRF20 filled with a CB content of 20 phr. Homogenous distributions of CB in the rubber matrix are observed in Figure 3a–c. As shown in Figure 3d, a large agglomeration of SRF CB occurs in the EPDM composite with a concentration of 20 phr. The CB filler shapes and distributions are identifiable in the visible TEM image exhibiting the CB filler particles on the rubber matrix. EPDM HAF20 and SRF20 exhibit spherical island shapes with polarized particle sizes of approximately 32 and 65 nm, respectively. The CB particles are distributed as partially condensed aggregates. The aspect ratio is defined by the ratios of the horizontal lengths to the vertical lengths of the particles. For EPDM HAF20 and SRF20 specimens with spherical shapes, the aspect ratios are 1.

Moreover, well-dispersed filler particles with small particle sizes on average, such as HAF CB filler, lead to similar results as filler particles with a high filler surface area and strong interactions with the polymer, thus affecting the gas diffusion and permeation processes. In HAF CB-filled EPDM composites crosslinked with sulfur, we measured the degree of filler dispersion according to the testing method (ASTM D7723). The measured dispersion degrees for two EPDM HAF20 and HAF60 were determined to be 98%; thus, these specimens are regarded as well-dispersed fillers in the rubber network. We did not find any remarkable differences in the dispersion degrees for samples with different filler contents.

### 4.2. Filler Effects on H_2_ Uptake

**Figure 4:**
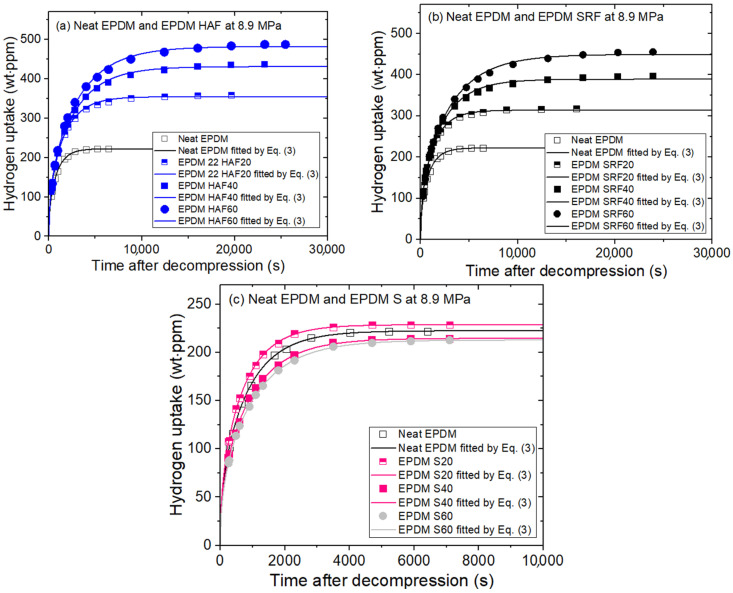
H_2_ uptake characteristics of the (**a**) EPDM HAF, (**b**) EPDM SRF, and (**c**) EPDM S series after hydrogen exposure at 8.9 MPa for 20 h and decompression. The solid lines are the least-squares fittings of Equation (3) using the diffusion analysis program. The results of neat EPDM are included in three panels for comparison with the EPDM composites blended with fillers.

The time evolution characteristics of H_2_ emission after decompression at pressures ranging from 1.2 to 90 MPa were measured in ten EPDM composites blended with CB and silica, and in neat EPDM. Figure 4 shows a plot of H_2_ uptake versus the elapsed time in ten EPDM rubbers after hydrogen exposure at 8.9 MPa for 20 h. The prominent characteristic is the increase in hydrogen uptake in CB-filled EPDM composites relative to that in neat EPDM. This phenomenon is attributed to H_2_ adsorption due to the presence of the CB filler. Increasing the HAF CB content in the HAF CB-filled EPDM composites increased the H_2_ emission content. The filler effect on the SRF CB-filled EPDM composites is similar to that of HAF CB-filled EPDM. The slight increase in H_2_ uptake for HAF CB-filled EPDM might be explained by the larger specific surface areas of the HAF CB filler compared with those of the SRF CB filler. In the silica-filled EPDM composites, the variation in H_2_ uptake with silica filler content is not obviously different from that of the neat EPDM polymer. This result implies that hydrogen is not adsorbed at the silica filler surface.

**Figure 5:**
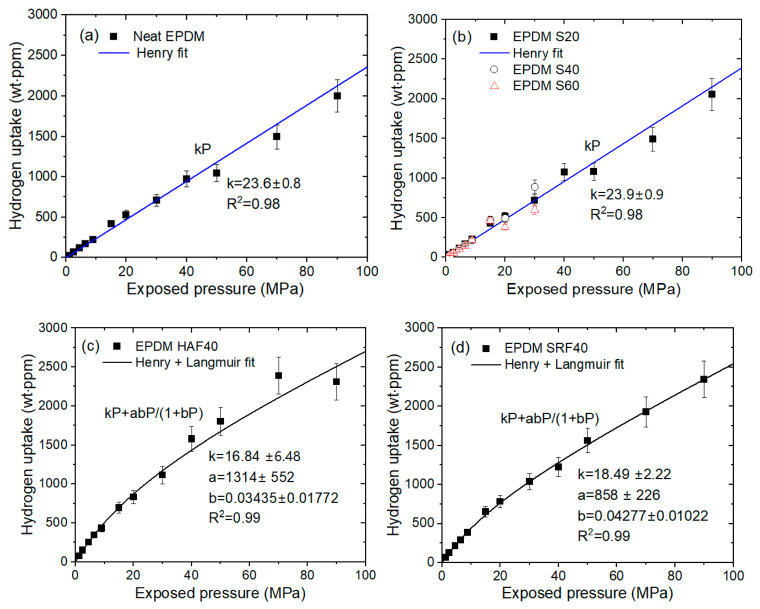
Relationship between H_2_ uptake ($C_{\infty }$) and exposure pressure for (**a**) neat EPDM, (**b**) EPDM S series, (**c**) EPDM HAF40, and (**d**) EPDM SRF40. The blue and black lines represent the Henry fit and the dual mode (Henry–Langmuir) fit, respectively. The legends show the linear least-squares fitting plots and their squared correlation coefficients (R^2^).

We measured the hydrogen emission content as a function of exposed pressure for nine EPDM composites blended with fillers and one neat EPDM. Figure 5 shows a plot of the representative hydrogen uptake data versus the pressure for four EPDM composites. Panels (a), (b), (c), and (d) of this figure display the pressure behaviors of H_2_ uptake with neat EPDM, EPDM composites compounded with silica filler, EPDM HAF40, and EPDM SRF40, respectively. All EPDM composites blended with HAF CB and SRF CB fillers reveal similar uptake behaviors versus pressure. To avoid redundancy, we only present the representative hydrogen uptake data for two CB-filled EPDM composites.

The H_2_ uptakes (*C*_∞_) of neat EPDM and EPDM S20 (Figure 5a,b) are proportional to pressures reaching 90 MPa, which is in accordance with Henry’s Law [33,34]. This behavior is responsible for the absorption of H_2_ into the polymer matrix. However, as shown in Figure 5c,d, the hydrogen uptakes for EPDM HAF40 and SRF40 deviate from Henry’s law at pressures above 15 MPa; this phenomenon is attributed to the adsorbed hydrogen at the surface of the CB filter. Thus, dual sorption is observed for all CB-blended EPDM composites. The dual mode sorption behaviors that cover the overall pressure range reaching 90 MPa are introduced as follows:

(4) $$C_{\infty }=kP+\frac{abP}{1+bP}$$

where *C*_∞_ is the total H_2_ gas uptake. The first term indicates Henry’s law with the Henry’s law coefficient *k*. The second term presents the Langmuir model [35,36], where a is the maximum adsorption quantity (or capacity parameter) and b is the adsorption equilibrium constant (or the Langmuir hole affinity parameter). The fitting results of the H_2_ uptake characteristics according to Equation (4) are summarized in Table 2.

**Figure 6:**
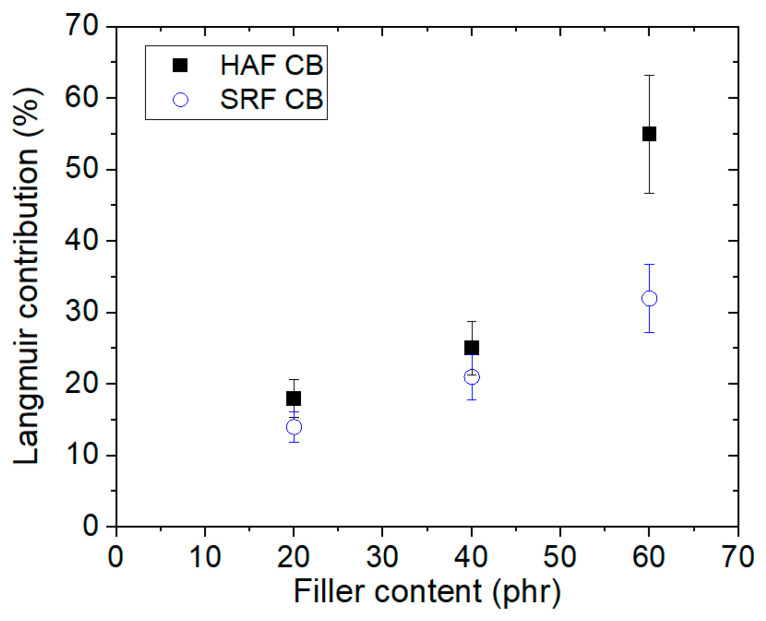
Langmuir contribution versus filler content for CB-filled EPDM composites.

The Langmuir contribution is obtained with respect to total hydrogen uptake, which is the uptake sum of Henry and Langmuir contributions. The Langmuir contribution indicates that the adsorption quantity of hydrogen increases with increasing filler content, as shown in Figure 6. The deviations from linearity above 60 phr for CB-filled EPDM composites indicate an abrupt increase in hydrogen adsorption; this phenomenon may be caused by the formation of hydrogen path channels and thus lead to a percolation effect by many fillers.

Langmuir sorption is related to the porous solids in the gas–polymer system. The Langmuir sorption site in a glassy polymer corresponds to holes or microvoids that arise due to the nonequilibrium nature of glassy polymers. A gas sorption isotherm in a glassy polymer below the glass transition temperature (T_g_) generally depends on the pressure exposure. This behavior is characteristic of dual-mode sorption with Henry’s law absorption in an equilibrium state and Langmuir adsorption in a nonequilibrium state [37]. The nonequilibrium state is directly related to the excess free volume or unrelaxed free volume in a glassy polymer [38]. Bondar et al. [39] confirmed the validity of dual mode behaviors. Therefore, the dual mode sorption model for gas sorption in glassy polymers is an effective method for investigation.

However, Jung et al. [25] demonstrated that, for HAF CB-filled NBR, the experimental data at the rubbery phase polymer show dual mode sorption due to the presence of porous HAF CB filler. H_2_ molecules are absorbed by rubbery NBR and are simultaneously adsorbed by porous filler, leading to dual mode sorption similar to that at the glass phase. Thus, the porous HAF CB filler in the NBR composite corresponds to the robust void structure in the glass phase polymer. The solubility result in HAF CB-filled NBR supports the dual sorption behavior.

### 4.3. Filler Effects on H_2_ Diffusion

Similar to the pressure-dependent H_2_ uptake, the H_2_ diffusivities of the neat EPDM and nine filled EPDM composites were measured as functions of exposed pressure. The H_2_ diffusivities of neat EPDM and the EPDM composites blended with fillers apparently depend on the exposed pressure. The pressure dependence of the diffusion coefficient is related to the decrease in the mean free path of H_2_, the increased tortuosity caused by the impermeable filler in the rubber networks and the increased interactions between the filler and the rubber.

**Figure 7:**
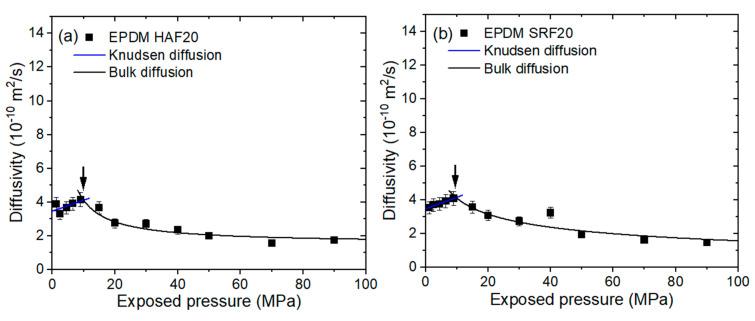

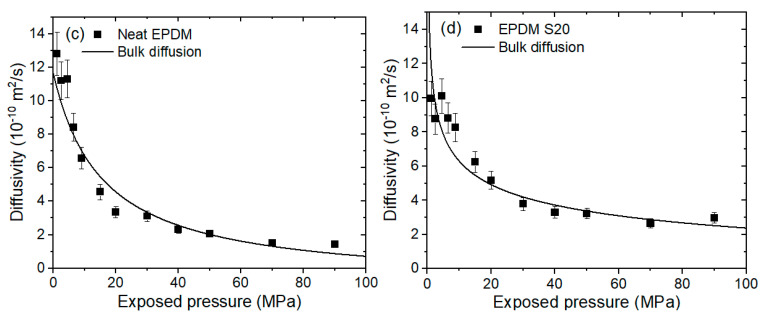
H_2_ diffusivity versus exposed pressure in (**a**) EPDM HAF20, (**b**) EPDM SRF20, (**c**) neat EPDM, and (**d**) EPDM S20. The blue lines indicate Knudsen diffusion fitted by Equation (5). The black lines indicate bulk diffusion fitted by Equation (6). The arrows in (**a**,**b**) indicate the intersecting regions of Knudsen and bulk diffusion.

All EPDM composites blended with HAF CB and SRF CB fillers revealed similar diffusion behaviors versus pressure. To avoid redundancy, the representative pressure-dependent diffusion for EPDM HAF20 and SRF20, as shown in Figure 7a,b, respectively, can be divided into two contributions at the peak, as indicated by arrows. The contributions correspond to Knudsen diffusion for low pressure and bulk diffusion for high pressure. The pressure-dependent behavior for diffusivity is interpreted by the results of Knudsen diffusion below 7–10 MPa, and bulk diffusion above this pressure range; this combined diffusion was observed and analyzed by fractal theory in other studies [40,41]. Knudsen diffusion gradually increases with increasing pressure. Knudsen diffusion below the pressure range normally occurs when there is a large mean free path of diffusing gas molecules or a low gas density. The Knudsen diffusion coefficient ($D_{K,\;pm}$) in porous media is expressed as follows [42]:

(5) $$D_{K,pm}=\frac{\emptyset }{\tau }D_{K}=\frac{\emptyset }{\tau }\frac{d_{c}}{3}\upsilon$$

where ϕ is the pressure-dependent porosity, τ is the tortuosity caused by introducing the filler, $d_{c}$ is the pore diameter, and υ is the average molecular velocity derived from the kinetic theory of gases.

Moreover, the bulk diffusion for neat EPDM (Figure 7c) and EPDM S20 (Figure 7d), and the bulk diffusion above a critical pressure of 7–10 MPa for CB-filled EPDM composites, are inversely proportional to pressure; this phenomenon is associated with the mean free path between the H_2_ molecules. Bulk diffusion is predominant when the mean free path (λ) in large pores is smaller than the pore diameters, or when high-pressure gas diffusion occurs. The bulk diffusion coefficient ($D_{B})$ is expressed as follows [43]:

(6) $$D_{B}=\frac{1}{3}\lambda \upsilon =\frac{1}{3}\frac{5}{8}\frac{\mu }{P}\sqrt{\frac{RT\pi }{2M}}\upsilon$$

where μ is the viscosity of the diffusion molecule in units of kg m/s and P is the pressure. The factor 5/8 considers the Maxwell–Boltzmann distribution of molecular velocity. The experimental results of the diffusivity shown in Figure 7 are fitted by both Equations (5) and (6), as indicated by the blue and black lines, respectively. In the region of Knudsen diffusion, the diffusion coefficient is proportional to the pressure; this phenomenon may be caused by an increase in the porosity in Equation (5) due to an increase in the pressure. The decrease in the bulk diffusion coefficient is attributed to a decrease in the mean free path with increasing pressure.

Regarding the H_2_ sorption and diffusion mechanism, we again justify the hydrogen sorption (diffusion) mechanism considering the role of CB. According to the hydrogen uptake data shown in Figure 5, the hydrogen sorption mechanisms in the EPDM composites blended with CBs (HAF and SRF) revealed two types of sorption (or diffusion): fast diffusion due to the hydrogen absorbed in the polymer network and slow diffusion due to the hydrogen physically adsorbed at the CB filler interface. In other words, the sorption (or diffusion) mechanism in CB-filled EPDM represents dual sorption (or diffusion) behaviors. In this study, the sorption and desorption processes of most H_2_ are reversible; this finding may be attributed to physisorption rather than chemisorption by penetrated H_2_.

However, a single hydrogen sorption (or diffusion) behavior in silica-filled EPDM is observed only with a fast-diffusing polymer network. The single-mode behavior is also shown in neat EPDM due to fast H_2_ sorption for the polymer network. Hydrogen in the silica-filled EPDM was not adsorbed at the interface between the silica and rubber. This finding indicates that hydrogen sorption in silica or at the interface between silica and the rubber matrix did not occur. Thus, as shown in the H_2_ uptake characteristics (Figure 5) for silica-filled EPDM specimens, the value (uptake) for silica-filled EPDM composites is nearly identical to that for neat EPDM. The result for CB-filled EPDM composites indicates that the fast component shows the permeation characteristics of H_2_ absorbed onto the parent component of the rubber (Henry’s law); the slow component shows the permeation characteristics of H_2_ adsorbed by the filler (Langmuir law).

**Figure 8:**
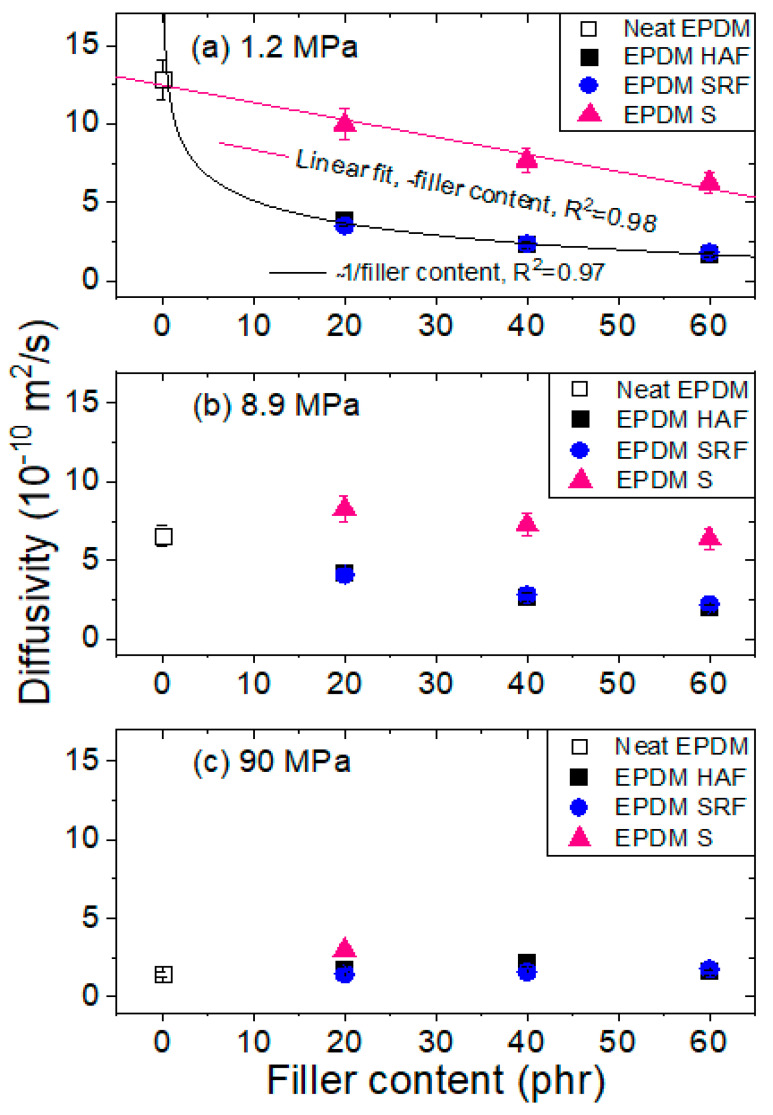
Diffusivity versus filler content at exposure to pressure of (**a**) 1.2 MPa, (**b**) 8.9 MPa, and (**c**) 90 MPa in EPDM composites blended with CB and silica. The pink line in (**a**) is fitted with a negative linear relationship between the diffusivity and the filler content. The black line in (**a**) is fitted with a linear relationship between the diffusivity and the reciprocal filler content. R^2^ is squared correlation coefficients of fitting.

Figure 8a–c shows the variations in the diffusivity characteristics with the filler content at the three different pressures of 1.2 MPa, 8.9 MPa, and 90 MPa, respectively. At a low pressure of 1.2 MPa, all fillers extend the diffusion path due to increased tortuosity by the impermeable filler, resulting in a decrease in the diffusion rate. The diffusivity in the silica-blended EPDM is negative and it decreases linearly with increasing filler content; the diffusivity in the CB-blended EPDM decreases in the form of an asymptotic line (~1/filler content). At the low pressure of 1.2 MPa, the decrease in diffusivity of the CB-blended EPDM is larger than that of the silica-blended EPDM; this phenomenon is expected and possibly related to the additional filler–polymer interactions. However, with the increasing pressure reaching 90 MPa, the filler effect on diffusion decreases; the diffusivity characteristics for all specimens converge at values of approximately 2 × 10^−10^ m^2^/s.

Two general models [18] are employed to explain the change in diffusivity by the presence of filler particles. These models differ in their descriptions of the interactions between filler particles and the polymer matrix. One model is based on the concept of free volume. Free volume ascribes the change in diffusivity to an increase or decrease in the microscopic friction coefficient of the diffusing species. This change is responsible for the influence of the filler surface on the mobilities of diffusing molecules in the vicinity of the filler particles through filler–polymer interactions. The second model is an obstacle model. Obstacles apparently decrease the diffusivity by increasing the tortuosity of the diffusion path or by creating bottlenecks without affecting the friction experienced by the diffusing species. A change in free volume can increase or decrease the polymer diffusivity; the presence of obstacles always decreases the polymer diffusivity. As shown in Figure 8a, the diffusivity for silica-filled EPDM is responsible for the tortuosity of the diffusion path by introducing filler (second model). Moreover, the diffusivity for CB-filled EPDM is attributed to both polymer–filler interactions and tortuosity (first and second model).

### 4.4. Correlations of Permeation with Density and Tensile Strength

**Figure 9:**
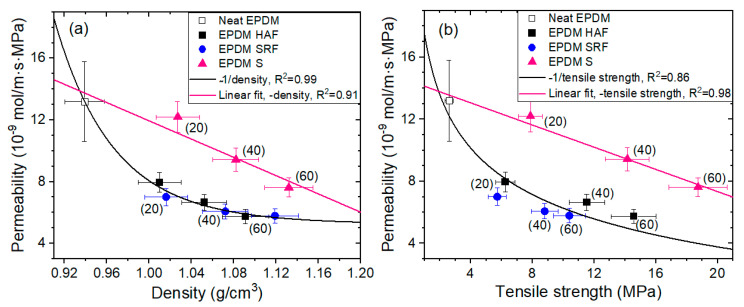
Correlations between permeability and (**a**) density and (**b**) tensile strength for neat EPDM and EPDM composites blended with CB/silica. The data for neat EPDM are included as pink and black curves for consistency with the fittings of the filler-blended EPDM composites. The value in parentheses indicates the phr of CB and silica. R^2^ is squared correlation coefficients of fitting.

The permeation *P* was determined by multiplying the solubility *S* by the diffusion coefficient *D*, i.e., *P* = *SD*. Figure 9a,b shows the permeability variations with density and tensile strength, respectively, for neat EPDM and blended EPDM polymer composites. The trends are similar to those of diffusivity at 1.2 MPa (Figure 8a), implying that permeation is predominantly affected by diffusivity rather than by solubility.

As shown in Figure 9a, the negative linear relationship (density) between permeability and density for silica-blended EPDM composites indicates a smooth decrease in permeation with increasing density, without introducing other interactions or additional parameters. However, the density effect on the permeability for CB-blended EPDM composites is inversely proportional to the filler content, i.e., ~1/density. The magnitude of the effects for the CB-blended EPDM composites is larger than that for the silica-blended EPDM composites. This result again implies an additional effect; that is, the polymer–filler interaction for CB-blended EPDM composites may originate the permeability behavior, as already shown in the pressure-dependent effect on diffusivity at 1.2 MPa. A similar behavior for the density influence on permeability was found in polyethylene gas permeability investigations with different permeants [44,45]. The decrease in permeability in polyethylene with increasing density is attributed to the volume dilution of the amorphous fraction by the relatively impermeable crystalline phase.

Moreover, the permeability changes with tensile strength shown in Figure 9b exhibit identical behaviors to the permeability changes with density, as shown in Figure 9a. The two trends may be closely related to the same origin. From the investigated relation function for physical and mechanical properties, we provide a possibility for predicting the H_2_-permeation properties of compounded EPDM candidates used as seal materials under high pressure in H_2_-refueling stations.

## 5. Conclusions

By using a volumetric analysis technique and an ungraded diffusion analysis program calculating up to a hundred summation terms in an expansion series of the concentration $C_{E}\left(t\right)$ of emitted H_2_, we investigated the H_2_-permeation characteristics of EPDM composites. The investigated results are summarized below.

The pressure-dependent H_2_ uptakes for neat EPDM and silica-filled EPDM composites show single sorption models that satisfy Henry’s law; this phenomenon was dominated by absorption by the polymer. The contribution from the filler was negligibly small. Moreover, H_2_ uptakes for CB-filled EPDM composites followed dual sorption models that obey Henry’s law and Langmuir law. The H_2_ uptake in the CB-filled EPDM received contributions from absorption by the polymer networks and adsorption by the CB filler. The difference between the two CBs is attributed to the distinct specific surface areas.

The diffusivity values in all EPDMs investigated depended on pressure. The decrease in the diffusivity for silica-filled EPDM relative to that for neat EPDM was responsible for the increase in the tortuosity of the diffusion path by introducing filler. Moreover, the decrease in the diffusivity for CB-filled EPDM was attributed to both polymer–filler interactions and tortuosity by impermeable fillers.

At 1.2 MPa, the silica-blended EPDMs show negative linear correlations between diffusivity and filler content. The relationship is very similar to that of permeability with the density and tensile strength characteristics of EPDM composites. The CB-blended EPDMs exhibit reciprocal relationships between diffusivity and filler content, likely for permeability with the density and tensile strength characteristics of EPDM composites. From the investigated relationships, we predicted the H_2_-permeation properties of compounded EPDM candidates when used as seal materials under high pressure in H_2_-refueling stations. In the present work, it was demonstrated that EPDM HAF60 and EPDM SRF60 specimens with large density and tensile strength characteristics exhibit the lowest H_2_ permeation among the specimens; these specimens are suitable candidates for high-pressure gas seals to prevent gas leakage.

## Figures and Tables

**Table 1:** Chemical compositions of sulfur-crosslinked EPDM rubber composites filled with HAF CB, SRF CB, and S fillers.

Composites	EPDM	ZnO	St/A	HAF N330	SRF N774	Silica S-175	Si-69	PEG	S	TBBS	MBT
Neat EPDM	100	3.0	1.0						1.5	1.0	0.5
EPDM HAF 20	100	3.0	1.0	20					1.5	1.0	0.5
EPDM HAF 40	100	3.0	1.0	40					1.5	1.0	0.5
EPDM HAF 60	100	3.0	1.0	60					1.5	1.0	0.5
EPDM SRF 20	100	3.0	1.0		20				1.5	1.0	0.5
EPDM SRF 40	100	3.0	1.0		40				1.5	1.0	0.5
EPDM SRF 60	100	3.0	1.0		60				1.5	1.0	0.5
EPDM S 20	100	3.0	1.0			20	1.6	0.8	1.5	1.0	0.5
EPDM S 40	100	3.0	1.0			40	3.2	1.6	1.5	1.0	0.5
EPDM S 60	100	3.0	1.0			60	4.8	2.4	1.5	1.0	0.5

**Table 2:** Fitting results of the sorption model for neat EPDM and EPDM rubber composites filled with HAF CB, SRF CB, and S fillers according to Equation (4).

Composites	k	a	B	R^2^	Langmuir Contribution (%)
Neat EPDM	23.6	0	0	0.98	0
EPDM HAF20	17.8	909	0.0315	0.99	18
EPDM HAF40	16.8	1314	0.0344	0.99	25
EPDM HAF60	5.18	1367	0.0547	0.98	55
EPDM SRF20	18.6	502	0.0498	0.99	14
EPDM SRF40	18.5	858	0.0428	0.99	21
EPDM SRF60	18.0	1528	0.0294	0.99	32
EPDM S20	23.9	0	0	0.98	0

## Data Availability

The data used to support the findings of this study are available from the corresponding author upon request.
